# Limited added value of systematic spinal cord MRI vs brain MRI alone to classify patients with MS as active or inactive during follow-up

**DOI:** 10.1007/s00415-025-13068-2

**Published:** 2025-04-05

**Authors:** Jérémy Hong, Malo Gaubert, Mathilde Lefort, Jean Christophe Ferré, Emmanuelle Le Page, Laure Michel, Pierre Labauge, Jean Pelletier, Jérôme de Seze, Françoise Durand-Dubief, François Cotton, Gilles Edan, Elise Bannier, Benoit Combès, Anne Kerbrat

**Affiliations:** 1 Univ Rennes, CHU Rennes, Service de radiologie, 35000 Rennes, France; 2 EMPENN research team, U1128, Univ Rennes, Inria, CNRS, Inserm, IRISA UMR 6074, Rennes, France; 3 Univ Rennes, EHESP, CNRS, Inserm, Arènes - UMR 6051, RSMS (Recherche sur les Services et Management en Santé) - U 1309, 35000 Rennes, France; 4 Univ Rennes, CHU Rennes, Service de Neurologie, CHU Pontchaillou, 2 RUE Henri Le Guilloux, 35000 Rennes, France; 5 Neurology department, Montpellier University Hospital, Montpellier, France; 6 Aix Marseille Univ, APHM, Pôle de Neurosciences Cliniques, MICeME, Marseille, France; 7 Aix Marseille Univ, CNRS, CRMBM, Marseille, France; 8 CIC Strasbourg INSERM 1434, Strasbourg University Hospital, Strasbourg, France; 9 Neurology Department, Lyon University Hospital, Lyon, France; 10 Department of Radiology, UMR 5220 & INSERM U1044, Lyon Sud Hospital, Hospices Civils de Lyon, France CREATIS - CNRS, University Claude Bernard Lyon 1, Lyon, France

**Keywords:** Spinal cord, MRI, Multiple sclerosis, Disease activity, Longitudinal study

## Abstract

**Background:**

The utility of systematic spinal cord (SC) MRI for monitoring disease activity after a multiple sclerosis (MS) diagnosis remains a topic of debate.

**Objectives:**

To evaluate the frequency of disease activity when considering brain MRI alone versus both brain and SC MRI and to identify factors associated with the occurrence of new SC lesions.

**Methods:**

We conducted a retrospective analysis of clinical and imaging data prospectively collected over 5 years as part of the EMISEP cohort study. A total of 221 intervals (with both brain and spinal cord MRI scans available at 2 consecutive time-points) from 68 patients were analysed. For each interval, brain (3D Fluid-Attenuated Inversion Recovery (FLAIR, axial T2 and axial PD) and SC MRI (sagittal T2 and phase-sensitive inversion recovery, axial T2*w and 3D T1) were reviewed to detect new lesions. Each interval was classified as symptomatic (with relapse) or asymptomatic. The baseline brain and SC lesion numbers were computed.

**Results:**

SC MRI activity without clinical relapse and/or brain MRI activity was rare (4 out of 221 intervals, 2%). The occurrence of a new SC lesion was associated with the number of brain lesions at baseline (OR = 1.002 [1.000; 1.0004],* p* = 0.015) and the occurrence of a new brain lesion during the interval (OR = 1.170 [1.041; 1.314], *p* = 0.009), but not with the baseline SC lesion number (*p* = 0.6).

**Conclusion:**

These findings support the current guidelines recommending routine disease monitoring with brain MRI alone, even in patients with a high SC lesion load.

**Supplementary Information:**

The online version contains supplementary material available at 10.1007/s00415-025-13068-2.

## Introduction

Spinal cord (SC) lesions are common in people with MS (pwMS), occurring in approximately 80% of the cases [1]. They are included in diagnosis criteria [2] and have a strong prognosis value early in the disease course [3, 4]. However, the role of SC MRI in monitoring disease activity and treatment response after diagnosis is still debated. This is mainly due to challenges in interpreting longitudinal SC MRI images, time (doubling), and therefore costs, and the idea that SC lesions are unlikely to be asymptomatic. Accordingly, SC MRI is currently not recommended routinely in the international guidelines, with the exception of specific clinical situations [5].

Nevertheless, prior studies [6–9] have reported that asymptomatic SC lesions occur in 15–30% of clinically stable pwMS and approximately 10% had asymptomatic SC lesions without asymptomatic brain lesions, implying that a proportion of patients with disease activity may be missed with brain MRI only. In contrast, another recent large study limited to cervical spinal cord had reported only 1.9% of pwMS with new isolated asymptomatic SC lesions during follow-up [10].

In addition to these conflicting results, several limitations can be identified. First, most of these studies were retrospective with SC MRI rarely performed routinely, potentially leading to an over-representation of patients with SC symptoms. Moreover, SC imaging protocols likely influenced detection rates. For example, axial sequences greatly increase the detection rate of SC lesions compared with sagittal sequences alone [11], and sequences such as phase-sensitive inversion recovery (PSIR) [12–14] have demonstrated a better detection rate, with better confidence of cervical SC MS lesions compared to sagittal T2 and short TI inversion recovery (STIR) sequences.

Taking into account these limitations, the present study focussed on a cohort of relapsing remitting MS (RRMS) patients in the first years after diagnosis, with prospective follow-up including systematic both brain and spinal cord MRI over a period of 5 years. Multisequence SC acquisitions were performed to optimise new SC lesions detection and a systematic reading of brain MRI using artificial intelligence (AI)-based tools as an aid to radiological reading was conducted [15, 16]. Our primary objective was to describe the number of intervals regarded as active when both brain and SC MRIs were considered versus when only brain MRI was analysed. We also investigated the factors associated with the occurrence of new brain or SC lesions to identify subpopulations of pwMS who might benefit from routine SC MRI monitoring. Finally, the prognostic value of presenting a new SC lesion compared to new brain lesion only on subsequent relapse and disability worsening was evaluated.

## Materials and methods

### Participants

We conducted a retrospective analysis of clinical and imaging data that were prospectively collected in the EMISEP multicentre research cohort (EMISEP, ClinicalTrials ID: NCT02117375 [17, 18]). The study was approved by the institutional review board and written informed consents were obtained from all participants. The main inclusion criteria in the EMISEP cohort were: relapsing–remitting multiple sclerosis (RRMS) diagnosis according to the 2010 criteria [19]; age between 18 and 45 years old; first relapse within 12 months prior the inclusion; initial MRI severity > 9 T2 brain lesions and/or initial myelitis documented on SC MRI; Expanded Disability Status Scale (EDSS) ≤ 3; no relapse and no corticosteroids within the month before inclusion. The patients were enrolled between 2014 and 2017, and underwent clinical and radiological assessments at baseline, 12, 24, 36 and 60 months. For the present study, we included patients from the initial EMISEP cohort with at least both brain and spinal cord MRI scans of sufficient quality (i.e. without significant artefacts compromising interpretation) available at 2 time-points during follow-up. Thus, of the 81 patients enrolled in the initial EMISEP cohort, 68 were included in the present study (centres: Marseille = 5, Montpelier = 11, Rennes = 44, Strasbourg = 6 and Toulouse = 2) with 221 intervals analysed (i.e. with both brain and spinal cord MRI scans available at two consecutive time-points. For each patient, MRI scans were paired with no repetition of the same initial time-point when more than two time-points were available).

### Clinical assessment and definitions

Neurologists specialising in MS conducted clinical evaluations every six months, recording relapse occurrence, disease-modifying therapy (DMT) use, and EDSS scores. A relapse was defined as the development of new or reactivation of pre-existing neurological symptoms lasting a minimum of 24 h in the absence of fever or infection, occurring at least 30 days after the preceding relapse. The relapses were confirmed by a supplementary visit with a clinical assessment performed by a MS specialist in the EMISEP study. An interval between two MRI scans during follow-up was considered as symptomatic if a relapse has occurred during this interval, and asymptomatic otherwise. The therapeutic strategy during each interval, and for each patient was defined as “under high efficacy therapy” (HET), “under medium efficacy therapy” (MET), “untreated”, or “switch between therapeutic strategies”. An interval “under HET” was defined as an interval under natalizumab, mitoxantrone, alemtuzumab or antiCD20 (between 3 months after treatment start and until treatment stop for natalizumab, and between 3 months after treatment start and until 1 year after treatment stop for the other HET). An interval “under MET” was defined as an interval under interferon, glatiramer acetate, teriflunomide, dimethyl Fumarate or fingolimod (between 6 months after treatment start and until treatment stop). An “untreated” interval was defined as an interval without any DMT. A “switch” interval corresponded to all the other periods. A disability worsening was defined as a confirmed and maintained until the last follow-up EDSS increase of 1.5 points for patients with baseline EDSS = 0, 1 point from 1 to 5.5, and 0.5 points for > 5.5.

### MRI assessment

MRI scans were performed using 3 T MRI scanners from the same manufacturer (Rennes, Strasbourg, Marseille: MAGNETOM Verio (VB17); Montpellier, Toulouse: MAGNETOM Skyra (VD13); Siemens Healthineers, Erlangen, Germany). Details of acquisition parameters are given in the Supplemental Material. Briefly, the same MR sequences and parameters were used across the centres: 1) for SC: sagittal T2-weighted TSE (T2w) and sagittal phase-sensitive inversion recovery (PSIR), with separate acquisitions for cervical and thoracic SC, and axial T2*-weighted (T2*w) and 3D T1-weighted (T1w) sequences from C1 to C7; 2) for the brain: axial T2-weighted, axial proton density (PD), and 3D fluid-attenuated inversion recovery (FLAIR).

### Image analysis

#### Baseline image analysis

A cross-sectional lesion segmentation was performed using deep-learning based automatic tools [20] for both brain (on 3D FLAIR) and spinal cord (on sagittal T2) acquisitions at baseline for each patient. SC lesion masks were systematically reviewed by JH, a radiology resident and manually adjusted if necessary.

#### Longitudinal image analysis

**Fig. 1:**
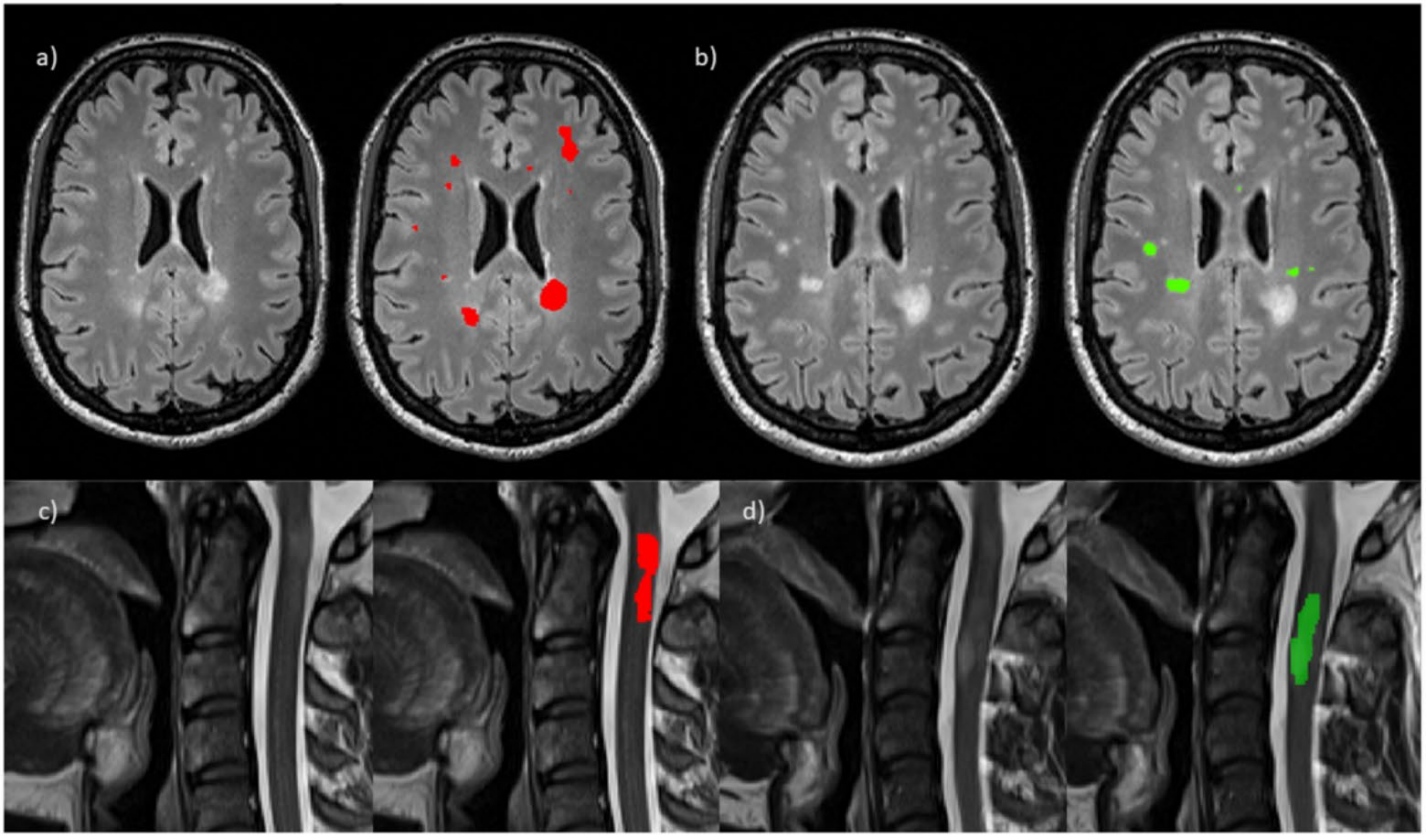
Examples of brain and spinal cord lesions segmentation at baseline and follow up (M12) in the same patient. **a** Baseline brain lesions segmentation on 3D FLAIR using a deep-learning based tool (red mask); **b** new brain lesion segmentation at follow-up identified with the help of a deep-learning based tool and registration (green mask); **c** baseline spinal cord lesions segmentation on sagittal T2 using a deep-learning based tool (red mask); **d** manual segmentation of a new spinal cord lesion segmentation at follow-up (green mask)

The 221 pairs of brain and spinal cord MRI scans were systematically reviewed by JH, blinded from the initial radiological reports performed by a neuroradiologist and from clinical information. For brain images, all available acquisitions (3D FLAIR, axial T2, and axial PD) were used to identify new/enlarging lesions, with the systematic help of a deep-learning-based tool (not used in initial reading) supplemented by registered 3D FLAIR volumes. The details regarding the deep-learning based tool use are available in [16]. The definition of “enlarging lesions” was left to the discretion of the radiologist, as is done in routine clinical practice, and in the absence of a precise definition in the current guidelines [5]. New/enlarging brain lesions were then reviewed and manually corrected, if necessary, by JH on the 3D FLAIR volume using ITK-SNAP software (version 4.0.1, [21]). For SC images, sagittal T2, PSIR, axial T2* and 3D T1 were used to identify new/enlarging lesions supplemented by registered sagittal T2 volumes. New/enlarging SC lesions were segmented on the sagittal T2 images, distinguishing the two acquisitions: “upper” and “lower”. These acquisitions (upper/lower) were an approximation of cervical and thoracic SC (the “upper” acquisition usually including a few thoracic vertebrae). In a second time, patient classification (as active (i.e. with new/enlarging T2 lesion) or inactive on brain and spinal cord MRI) was compared to the initial classification based on the initial radiological report. In case of discrepancies, images were reviewed by JH and another reader (AK, neurologist with 10 years of experience in interpreting brain and spinal cord MRI in pwMS) to correctly classify the patient (Fig. 1). Number and volume of cross-sectional lesions and new lesions between two time-points were extracted using FSL software (version 6.0.5, [22]) (fslstats and cluster).

### Statistical analysis

Baseline characteristics of the study population and the occurrence of new brain and spinal cord lesions during symptomatic and asymptomatic intervals were described using means with standard deviations for continuous variables and frequencies with percentages for categorical variables. For some non-normally distributed variables, medians with interquartile ranges (IQRs) were displayed.

Univariate logistic regression was first conducted to assess potential factors associated with the occurrence of new brain or spinal cord lesions (sex (ref. Women), age, MS duration, therapeutic strategy (ref. HET), relapses (year before), EDSS, number of SC lesion(s), number of brain lesion(s), new SC lesion(s), new brain lesion(s)). Variables with a *p*-value < 0.20 in the univariate analysis were then included in the final multivariable logistic regression. A *p*-value of less than 0.05 was considered statistically significant.

The time to first relapse or disability progression for patients with new brain or spinal cord lesions between groups were studied using Kaplan–Meier method with the corresponding log-rank test.

Given that multiple scans were obtained per patient over time, a patient effect was applied to account for the correlation between repeated measurements within the same patient. The analyses were performed using R (version 4.2.3).

## Results

### Baseline and follow-up patients’ characteristics

The baseline demographical, clinical and imaging characteristics of the population are detailed in Table 1. A total of 68 individuals with RRMS were included with a mean age of 31.5 years (SD = 6.4) at first MRI acquisition. Overall, pwMS had a short disease duration (mean = 8.2 months, SD = 4 months), a low EDSS score (median = 1 [IQR = 0–2]) and a wide range of brain and spinal cord lesion number (median = 22, IQR = 8–37 and 3, IQR = 2–6, respectively).

**Table 1:** Baseline characteristics of the study population

	People with RRMS
	*N* = 68
Gender—*F* (%)	44 (64.7)
Age (years)—mean (SD)	31.5 (6.4)
MS duration (months)—mean (SD)	8.2 (4,0)
Annual relapse rate from MS onset to baseline—mean (SD)	0.5 (0.3)
EDSS at inclusion—*N* (%)	
0	29 (42.6)
1	21 (30.9)
1.5	5 (7.4)
2	6 (8.8)
2.5	4 (5.9)
3–4	3 (4.4)
Volume of brain lesions (mm^3^)—median [IQR]	2164.4 [644.7, 5030.9]
Number of brain lesions—median [IQR]	22.0 [8, 37]
Volume of spinal cord lesions (mm^3^)—median [IQR]	455.75 [181.2, 951.2]
Number of spinal cord lesions—median [IQR]	3.0 [2.0, 6.0]

*%* percentage, *SD* standard deviation, *N* number, *IQR* interquartile range, *EDSS* Expanded Disability Status Scale

Over the 5-year follow-up, 221 intervals from the 68 patients were analysed. The mean number of intervals per patient was 3.25 (1.1) and the mean follow-up duration was 4.7 (0.99) years (with a mean duration of 1.4 years for each interval). The temporal distribution of the intervals across sites is detailed in Supplementary Table 1. Of these 221 intervals, 42 (19%) were classified as symptomatic (i.e. with the occurrence of at least 1 relapse during the interval), whilst 179 (81%) were asymptomatic. Regarding therapeutic strategies, 114 intervals were considered as a period of treatment by MET (52%), 16 by HET (7%), 55 as untreated (25%) and 30 as a period of switch between therapy strategies (14%). Nine out of 68 patients experienced a significant EDSS increase.

### New brain and spinal cord lesions during follow-up

Overall, when both symptomatic and asymptomatic intervals were considered, 97 out of 221 intervals (44%) had no new brain or SC lesion, 82 (37%) had only new brain lesion, 8 (4%) had only new SC lesion and 34 (15%) had both new brain and SC lesion. Amongst the 34 intervals with both new brain and SC lesions, the majority (25 out of 34) involved at least three new brain lesions.

**Fig. 2:**
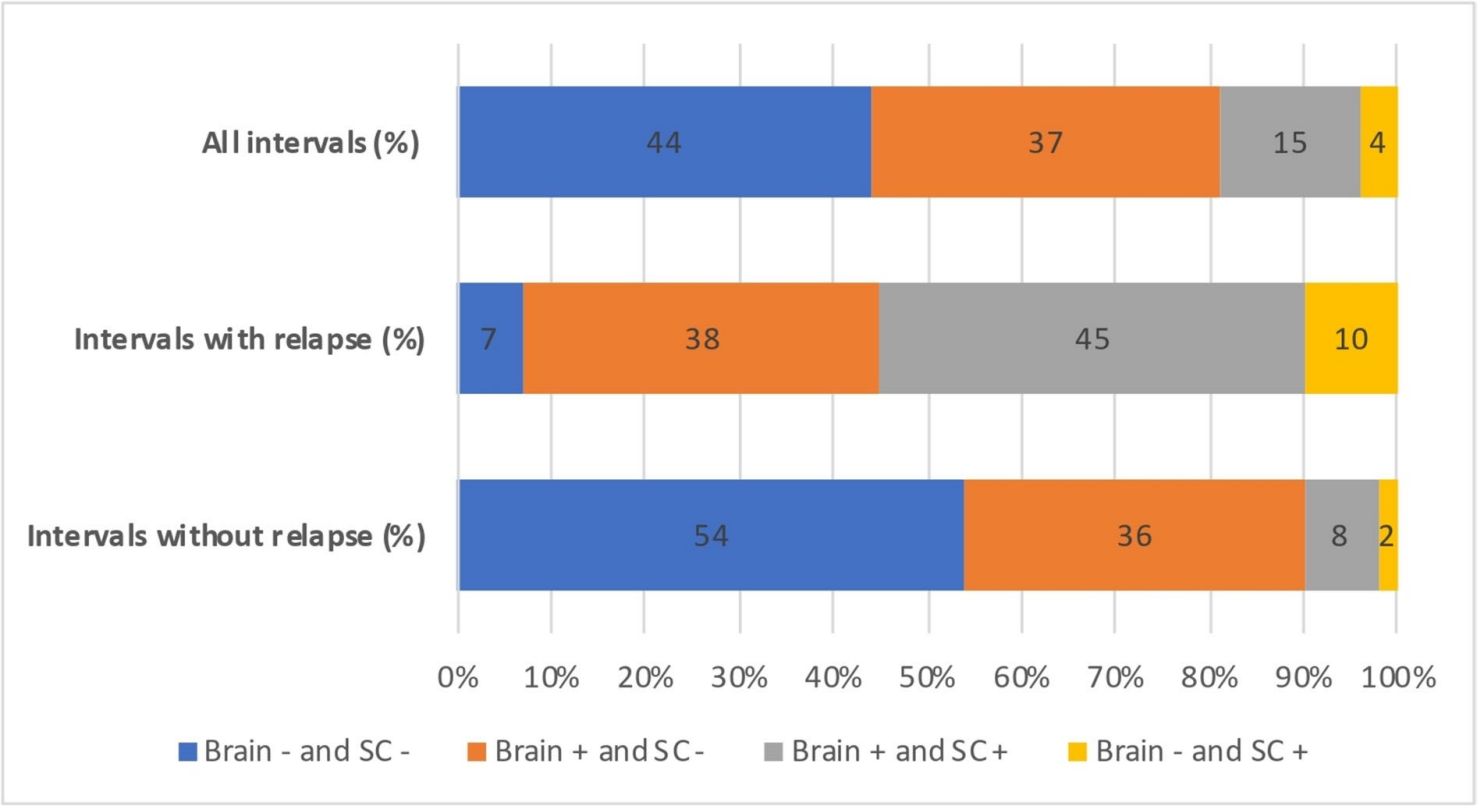
Brain and spinal cord MRI activity according to clinical activity. Percentage of MRIs with brain and/or spinal cord new lesions. Brain+ : interval with new brain lesion; Brain−: interval without new brain lesion; SC+ : interval with new spinal cord lesion; SC−: interval without new spinal cord lesion

When only asymptomatic intervals were considered (*n* = 179), 4 intervals (2%) had isolated new SC lesions and 15 (8%) had both new brain and SC lesions. Of the 15 intervals with both new brain and SC lesions, the majority had at least three new brain lesions (10 out of 15). The results were very different when only symptomatic intervals were considered (*n* = 42), with 4 intervals (10%) with isolated new spinal cord lesions and 19 (45%) with both new brain and spinal cord lesions. These results are summarised in Fig. 2.

### New cervical vs. thoracic spinal cord lesions during follow-up

The occurrence of new cervical cord lesions was higher than thoracic lesions during follow-up. Of the 42 intervals with SC MRI activity (out of 221), 28 had one or more new cervical spinal cord lesions with no thoracic lesions (67%), six had both new cervical and thoracic lesions (14%), and only eight had one or more new thoracic lesions without new cervical lesions (19%).

### Factors associated with the occurrence of new brain or new spinal cord lesions

In the multivariable analyses, the occurrence of a new SC lesion was positively associated with period of treatment switch (compared to periods under HET), with the number of brain lesions on the initial MRI scan of the interval and with the occurrence of new brain lesion(s) during the same interval (Table 2). No significant association was found with the number of SC lesions on the initial MRI scan of the interval.

**Table 2:** Factors associated with the occurrence of a new spinal cord lesion

	Univariate	*p*	Multivariable	*p*
	OR [95%IC]		OR [95%IC]	
Sex (ref. women)	0.992 [0.890; 1.106]	0.889		
Age	0.996 [0.988;1.004]	0.318		
Disease duration	0.977 [0.934; 1.022]	0.303		
Treatment (ref. HET)		**0.003**		**0.006**
MET	0.998 [0.814; 1.223]		1.145 [0.909; 1.443]	
SWITCH	1.316 [1.039;1.668]		1.385 [1.061; 1.808]	
UNTREATED	1.138 [0,916; 1.414]		1.221 [0,960; 1.553]	
Relapse (year before)	1.043 [0,979; 1.111]	**0.189**	1.049 [0.983; 1.119]	0.147
EDSS	0.988 [0,938; 1.041]	0.655		
Nb of SC lesion(s)	1.003 [0.990; 1.016]	0.623		
Nb of brain lesion(s)	1.003 [1.001; 1.005]	**0.0003**	1.002 [1.000; 1.004]	**0.015**
New brain lesion(s)	1.246 [1.127; 1.378]	**< 10**^**–4**^	1.170 [1.041; 1.314]	**0.009**

*OR* odd ratio, *HET* high efficacy therapy, *MET* medium efficacy therapy, *Nb *number, *SC* spinal cord, *EDSS* Expanded Disability Status Scale

*p*-values<0.20 in the univariate analysis are shown in bold

*p*-values<0.05 in the multivariate analysis are shown in bold

In the multivariable analyses, the occurrence of a new brain lesion was positively associated with the periods of treatment switch compared to periods under HET, and with the occurrence of new spinal cord lesion(s) during the same interval (Table 3). Again, no significant association was found with the number of spinal cord lesions on the first MRI.

**Table 3:** Factors associated with the occurrence of a new brain lesion

	Univariate	*p*	Multivariable	*p*
	OR [95%IC]		OR [95%IC]	
**Sex (ref. women)**	0.960 [0.835; 1.101]	0.550		
Age	0.986 [0.976; 0,996]	**0.005**	0.990 [0.979; 1.002]	0.101
MS duration	0.915 [0.865; 0.967]	**0.002**	0.957 [0.860; 1.064]	0.411
Treatment (ref. HET)		**0.002**		**0.022**
MET	1.154 [0.893; 1.492]		1.272 [0.943; 1.715]	
SWITCH	1.628 [1.210; 2,192]		1.555 [1.103; 2.194]	
UNTREATED	1.285 [0,979; 1.688]		1.299[0,949; 1.779]	
Relapses (year before)	1.140 [1.054; 1.234]	**0.001**	1.083 [0.971; 1.206]	0.150
EDSS	1.026 [0.959; 1.097]	0.459		
Nb of SC lesion(s)	1.011 [0.994; 1.029]	0.194	1.003 [0.985; 1.020]	0.761
Nb of brain lesion(s)	1.003 [1.001; 1.006]	**0.011**	1.002 [0.999; 1.004]	0.203
New SC lesion(s)	1.429 [1.214; 1.682]	**< 10**^**–4**^	1.288 [1.060; 1.566]	**0.011**

OR odd ratio, HET high efficacy therapy, MET medium efficacy therapy, Nb number, SC spinal cord, EDSS Expanded Disability Status Scale

*p*-values<0.20 in the univariate analysis are shown in bold

*p*-values<0.05 in the multivariate analysis are shown in bold

#### Impact of new spinal cord lesions compared to new brain lesion only on the time to first relapse or disability progression

**Fig. 3:**
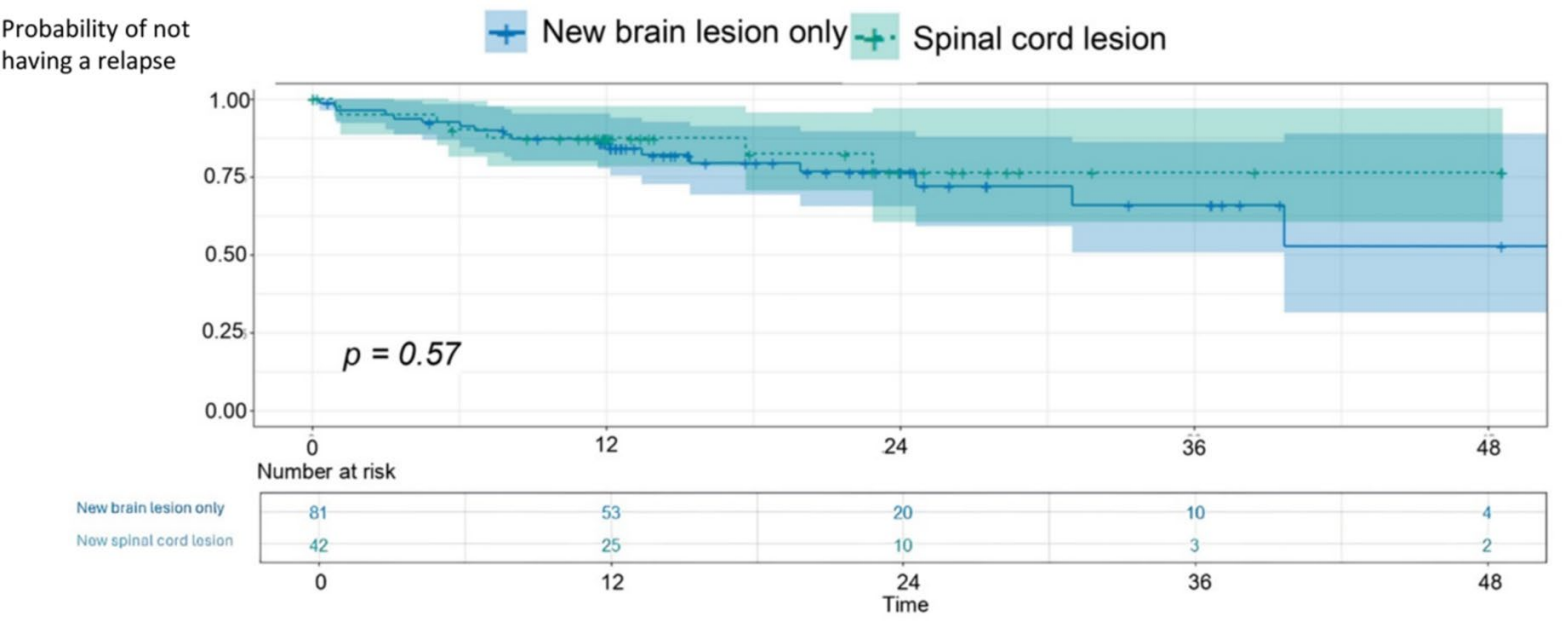
Time (months) to first relapse in patients with a new spinal cord lesion compared to patients with only new brain lesions

The time to first relapse was not significantly shorter in patients with at least a new SC lesion compared to patients with at least a new brain lesion only (*p* = 0.57, Fig. 3), whilst the time to first relapse was significantly decreased in patients with MRI activity (brain or SC) compared to those without MRI activity (*p* = 0.0039).

The time to disability progression was not significantly shorter in patients with new SC lesions compared to patients with new brain lesions only (*p* = 0.64, supplementary figure). However, only 10 out of 68 patients experienced an EDSS significant increase during follow-up.

## Discussion

In this retrospective study of a prospective cohort of early RRMS patients undergoing systematic brain and SC MRI follow-up according to a predetermined schedule for 5 consecutive years, we observed that SC MRI activity without concurrent clinical relapse and/or brain MRI activity was rare. The occurrence of new SC lesions was mainly associated with the number of brain lesions at baseline and the development of new brain lesions during follow-up, but not with the SC lesion load. Furthermore, the risk of relapse or disability progression following the detection of new SC lesions was not significantly increased compared to cases with only new brain lesions.

Our study highlighted a low frequency (2%) of isolated new SC lesions in asymptomatic patients, consistent with a recent study limited to the cervical SC, which found a risk of 1.9% [10] but contrasting with earlier studies [6–9] that reported higher rates (around 10%). Several factors may explain our results. First, unlike previous studies, patients included in the present study were followed prospectively according to a predetermined MRI schedule. Retrospective designs often introduce selection bias, as SC MRIs in addition to brain MRIs are typically performed at the discretion of neurologists, often in patients with symptoms suggestive of SC involvement. Second, a deep learning tool to help new brain lesion detection was used, as it is increasingly the case in routine clinical practice. A semi-automated co-registration fusion software for comparing FLAIR sequence was also used in Lim et al. [10]. These tools improve the detection of brain lesions, potentially reducing the proportion of patients classified as having isolated SC lesions by identifying concurrent brain lesions more accurately [23, 24]. The increasingly widespread use of HET in recent years could also have an impact on the risk of new SC lesions in recent studies. However, the majority of our cohort was treated with MET (as in the study by Lim et al.). A more recent cohort predominantly treated with HET might demonstrate an even lower rate of SC activity. Interestingly, our results were obtained using a protocol that included several SC sequences (sagittal T2, sagittal PSIR, axial T2* and 3DT1) and a double reading of the images. Thus, under real-world clinical conditions, the detection of SC lesions might be even lower. Our findings reinforce the current recommendations advocating for monitoring treatment efficacy based on brain MRI alone, unless symptoms suggestive of SC are present [5].

Moreover, in our study, the number of baseline SC lesions was not associated with the appearance of new SC lesions during follow-up, whilst the number of baseline brain lesions and the presence of new brain lesions were. This association between SC and brain MRI activity has been reported in previous studies [10, 25] and suggests that the overall inflammatory activity of a given patient plays a more significant role in the development of new SC lesions than the initial SC involvement itself. It is worth noting that current international guidelines recommend systematic SC MRI follow-up in patients with predominantly SC phenotypes [5]. Thus, further studies are needed to assess the risk of new SC versus brain lesions in patients with isolated SC lesions or a predominance of spinal cord lesions (i.e. with only a few brain lesions).

Finally, in our study, the risk of new clinical relapses during follow up was associated with MRI activity during a previous interval (regardless of lesion location). However, no significant difference was observed between patients with new cerebral lesions only and those with new SC lesions. This result is in line with previous studies which also showed that new SC lesions, independently of new brain lesions, were not associated with shorter time to first relapse [6, 8, 10].

Our study has several limitations that should be considered. First, our cohort is limited to 68 patients, although a total of 221 intervals were analysed. Second, our SC acquisition protocol was more detailed (four sequences) than what is routinely performed which may limit generalizability to clinical practice as this might have led to a higher detection rate of SC lesions. On the contrary, although the number of spinal cord sequences used in this study is substantial, we cannot exclude that the use of recent sequences such as MP2RAGE [26] or the use of 7 T MRI [27] would not have resulted in a higher detection rate of new spinal cord lesions. Spinal cord lesions are indeed difficult to detect, and similar studies, conducted with new, potentially more sensitive sequences, are yet to be carried out. Furthermore, in contrast to other studies [6–10, 28], our research protocol did not include post-gadolinium injection imaging. However, previous studies report no clear added value of gadolinium injection on new spinal cord lesion detection [6, 28]. In addition, the inclusion criteria of our study, which focussed on patients in the early stages of the disease with significant baseline brain or SC lesion loads, selected a subpopulation particularly at risk of MRI activity. However, these biases have likely led to an overestimation of SC activity compared to a more general MS population, and therefore, strengthens our conclusion regarding the low added value of routine SC MRI. Finally, our patient cohort remained relatively stable in terms of disability over the 5 years of follow-up. The prognostic value of the occurrence of new asymptomatic SC lesions versus the occurrence of new brain lesions could therefore not be accurately assessed, and would have required a larger cohort with longer follow-up.

In conclusion, we found that the proportion of new isolated SC lesions in asymptomatic pwMS was low (2%) and that the occurrence of a new SC lesion was strongly associated with the occurrence of concurrent new brain lesions. These findings support the current guidelines recommending routine disease monitoring with brain MRI alone, even in patients with high SC lesion loads, unless specific clinical indications for SC imaging are present.

## Supplementary Information

Below is the link to the electronic supplementary material.

Supplementary file1 (DOCX 2687 KB)

## Data Availability

The raw data supporting the findings of this study are available at https://osf.io/rew7f/
