# Supplementary material for: Limited added value of systematic spinal cord MRI vs brain MRI alone to classify patients with MS as active or inactive during follow-up
Source: J Neurol. 2025 Apr 5;272(4):316. doi: 10.1007/s00415-025-13068-2 (PMC11972184; doi:10.1007/s00415-025-13068-2)

**Limited added value of systematic spinal cord MRI vs brain MRI alone to classify patients with MS as active or inactive during follow-up**

Jérémy Hong^1^, Malo Gaubert^1,2^, Mathilde Lefort^3^, Jean Christophe Ferré^1^, Emmanuelle Le Page^4^, Laure Michel^4^, Pierre Labauge^5^, Jean Pelletier^6,7^, Jérôme de Seze^8^, Françoise Durand-Dubief^9^, François Cotton^10^, Gilles Edan^4^, Elise Bannier^2^, Benoit Combès^2^ and Anne Kerbrat^2,4,*^

^1^Univ Rennes, CHU Rennes, Service de radiologie, F-35000 Rennes, France

^2^EMPENN research team, U1128, Univ Rennes, Inria, CNRS, Inserm, IRISA UMR 6074

^3^Univ Rennes, EHESP, CNRS, Inserm, Arènes - UMR 6051, RSMS (Recherche sur les Services et Management en Santé) - U 1309 – F-35000 Rennes, France

^4^Univ Rennes, CHU Rennes, Service de neurologie, F-35000 Rennes, France

^5^Montpellier University Hospital, Neurology department, France

^6^Aix Marseille Univ, APHM, Pôle de Neurosciences Cliniques, MICeME, Marseille, France

^7^Aix Marseille Univ, CNRS, CRMBM, Marseille, France

^8^Strasbourg University Hospital, France, CIC Strasbourg INSERM 1434

^9^Lyon University Hospital, Neurology department, France

^10^Department of radiology, Lyon Sud Hospital, Hospices Civils de Lyon, France CREATIS - CNRS UMR 5220 & INSERM U1044, University Claude Bernard Lyon 1, Lyon, France

* **Corresponding author :** Anne Kerbrat ; address: Service de Neurologie, CHU Pontchaillou, 2 rue Henri Le Guilloux, 35033 Rennes cedex – France; email: anne.kerbrat@chu-rennes.fr; phone : +33 6 16 92 65 81

List of supplementary material

[Parameters of MRI sequences](#_t96qjtn3j8ns)

[Supplementary table 1. Temporal distribution of available MRI intervals](#_bmeezbx1drrx)

Supplementary Figure: Time to first disability progression in patients with a new spinal cord lesion compared to patients with only new brain lesions

#

# Parameters of MRI sequences

|  | **Acquisition Time (min)** | **Orientation** | **Alignment** | **Voxel size** | **TR (ms)** | **TE (ms)** | **TI (ms)** | **Flip angle (°)** | **FoV** | **Matrix size** | **Parallel Imaging** |
| --- | --- | --- | --- | --- | --- | --- | --- | --- | --- | --- | --- |
| **Brain** |  |  |  |  |  |  |  |  |  |  |  |
| Axial T2-weighted | 2:12 | Axial | AC-PC | 0.7x0.7x3 | 6530 | 9.4/84 |  | 150 | 220x220 | 320x320 | GRAPPA 2 |
| 3D FLAIR | 5 | Sagittal | AC-PC | 1×1×1.1 | 5000 | 399 | 1800 |  | 256×256 | 256×256 | GRAPPA2 |
| **Spinal cord** |  |  |  |  |  |  |  |  |  |  |  |
| Sagittal T2 (twice) | 3 | Sagittal | Cervical and dorsal | 0.9×0.7×2.5 | 3000 | 68 |  | 180 | 260×260 | 384x384 | GRAPPA2 |
| Axial T2* MEDIC (twice) | 4 | Axial | Perpendicular to the cord, C1-C3 and C4-C7 | 0.7×0.7×3 | 849 | 23 |  | 30 | 180×180 | 256×256 | GRAPPA2 |
| Sagittal phase-sensitive inversion recovery (PSIR) (twice) | 2:37 | Sagittal | Cervical and dorsal | 0.8x0.8x2.5 | 4585 | 9.8 | 400 | 160 | 260x260 | 320x320 | GRAPPA 2 |
| 3D T1-weighted (T1w) | 4:12 | Sagittal | From C1 to C7 | 1x1x1 | 1800 | 2.79 | 900 | 9 | 250x250 | 256x256 | GRAPPA |

*Description of parameters of all MRI sequences (brain and spinal cord) acquired in the study. Legend : MPRAGE = Magnetization-prepared rapid acquisition with gradient echo; FLAIR = Fluid-attenuated inversion recovery; MEDIC = Multiple echo data image combination; GRAPPA = Generalised autocalibrating partially parallel acquisitions; AC-PC = Anterior commissure - posterior commissure; TR = Repetition time; TE = Echo time; TI = Inversion time; FoV = Field of view.*

# Supplementary table 1: temporal distribution of available MRI intervals across sites
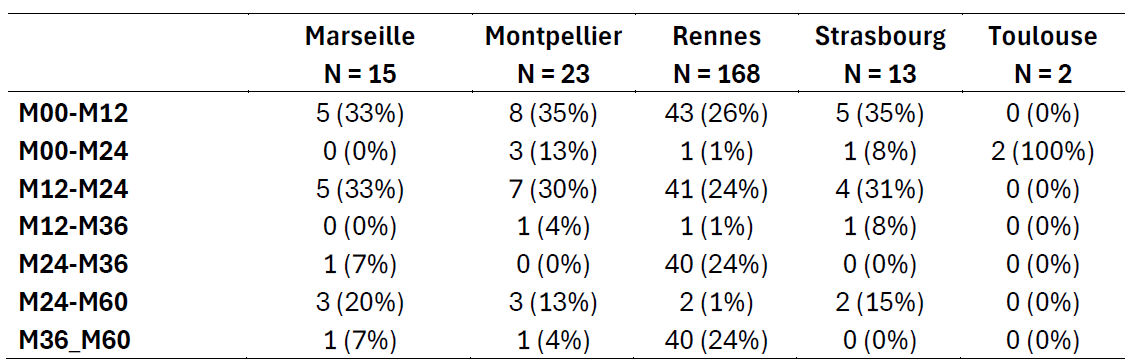


# Supplementary Figure: Time to first disability progression in patients with a new spinal cord lesion compared to patients with only new brain lesions


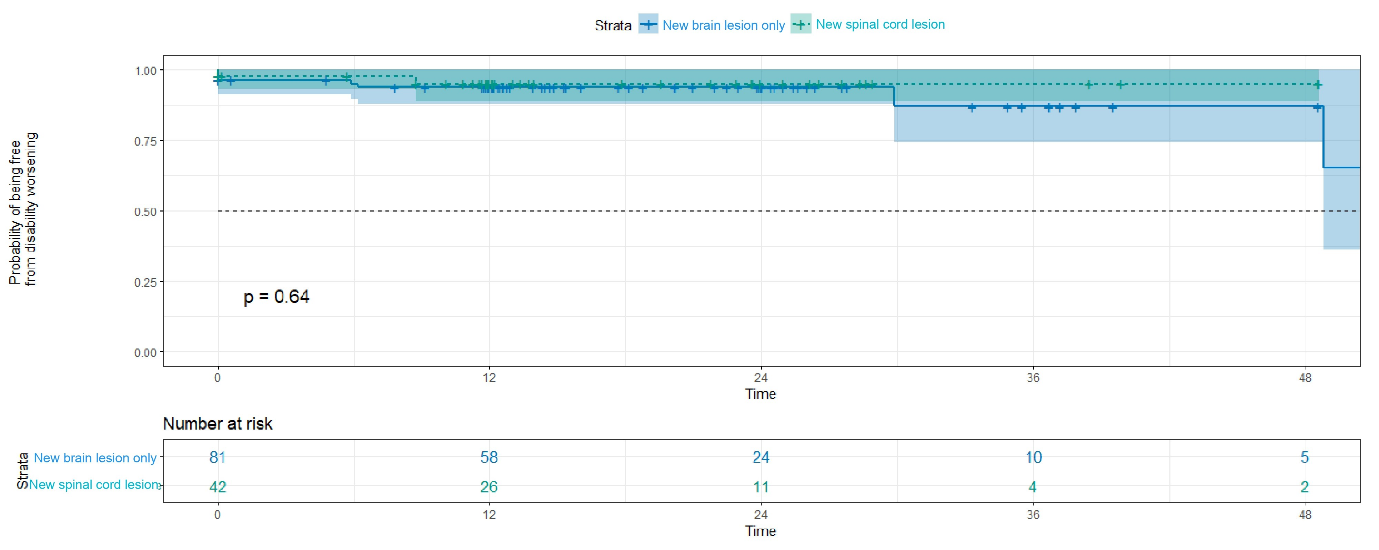

Supplement: Supplementary file 1 — Supplementary file1 (DOCX 2687 KB) [file 415_2025_13068_MOESM1_ESM.docx]
